# Inflammation triggers specific microRNA profiles in human adipocytes and macrophages and in their supernatants

**DOI:** 10.1186/s13148-015-0083-3

**Published:** 2015-04-24

**Authors:** Francisco José Ortega, María Moreno, Josep María Mercader, José María Moreno-Navarrete, Núria Fuentes-Batllevell, Mònica Sabater, Wifredo Ricart, José Manuel Fernández-Real

**Affiliations:** Department of Diabetes, Endocrinology and Nutrition (UDEN), Institut d’Investigació Biomédica de Girona (IdIBGi), Avinguda de França s/n, 17007 Girona, Spain; CIBER de la Fisiopatología de la Obesidad y la Nutrición (CIBERobn, CB06/03) and Instituto de Salud Carlos III (ISCIII), Sinesio Delgado 4, 28029 Madrid, Spain; Joint BSC-CRG-IRB program on Computational Biology, Barcelona Supercomputing Center, Baldiri Reixac 10, 08028 Barcelona, Spain

**Keywords:** microRNAs, RT-PCR, Profiling, Macrophages, Adipocytes, Adipose tissue, Obesity, Inflammation

## Abstract

**Background:**

The relevance of microRNAs (miRNAs) in adipose tissue is increasingly recognized, being intrinsically linked to different pathways, including obesity-related inflammation. In this study, we aimed to characterize the changes induced by inflammation on the miRNA pattern of human adipocytes and macrophages. Therefore, an extensive profile of 754 common miRNAs was assessed in cells (human primary mature adipocytes, and the macrophage-like cell line THP-1) and in their supernatants (SN) using TaqMan low-density arrays. These profiles were evaluated at the baseline and after administration of lipopolysaccharide (LPS, 10 ng/ml) and LPS-conditioned medium from M1 macrophages (MCM, 5%). The miRNAs that experienced the most dramatic changes were studied in subcutaneous human adipose tissue before and approximately 2 years after bariatric surgery-induced weight loss.

**Results:**

Differentiated adipocytes expressed 169 miRNAs, being 85 detectable in the SN. In M1 macrophages, 183 miRNAs were detected, being 106 also present in the SN. Inflammation led to an increased number of miRNAs detectable in cells and in their SNs in both adipocytes (+8.3% and +24.7%) and M1 macrophages (+1.4% and +5%, respectively). Indeed, under inflammatory conditions, adipocytes and M1 macrophages shared the expression of 147 (+9%) miRNAs, and 100 (+41%) common miRNAs were found in their SNs. Twelve of these factors were also linked to inflammation in whole adipose tissue from obese subjects. Interestingly, miR-221 (2-fold, *P* = 0.002), miR-222 (2.5-fold, *P* = 0.04), and miR-155 (5-fold, *P* = 0.015) were increased in inflamed adipocytes and in their SNs (15-, 6-, and 4-fold, respectively, all *P* < 0.001). Furthermore, their expressions in human adipose tissue concordantly decreased after weight loss (−51%, *P* = 0.003, −49%, *P* = 0.03, and −54.4%, *P* = 0.005, respectively).

**Conclusions:**

Inflammation induces a specific miRNA pattern in adipocytes and M1 macrophages, with impact on the physiopathology of obesity-induced inflammation of adipose tissue. The crosstalk between cells should be investigated further.

**Electronic supplementary material:**

The online version of this article (doi:10.1186/s13148-015-0083-3) contains supplementary material, which is available to authorized users.

## Background

Obese adipose tissue (AT) is a critical player in chronic low-grade systemic inflammation, with impact on insulin resistance through the secretion of hormones, adipocytokines [1], fatty acids [2], and (potentially) microRNAs [3] that modulate different metabolic pathways in AT itself, liver, skeletal muscle, and in the vasculature [4]. Differentiated adipocytes release a great amount of inflammatory cytokines and chemokines (the so-called adipokines) in response to activated macrophages [5,6]. Weight loss is known to result in reduced macrophage infiltration in parallel to decreased expression of pro-inflammatory genes [7,8]. Many of these factors are able to maintain sustained subclinical inflammation in obese AT, and activate other immune cells [9,10].

MicroRNAs (miRNAs) are non-coding RNAs that modulate gene expression through translational repression of specific target genes [11]. Many of these small RNAs are found in human AT, being differentially expressed in obese AT [12]. The close association between the miRNA expression profile in tissues and metabolic diseases such as obesity and type 2 diabetes is being increasingly recognized [13]. Several miRNAs are involved in adipocyte differentiation [14] and fat cell behavior [15]. So far, conditional disruption of Dicer, a key miRNA processing, may lead to impaired adipogenesis and fat mass shrinkage [16], reinforcing the relevance of miRNAs in AT development, inflammation, and metabolism. Not only tissue miRNAs play an important role, since specific circulating miRNA profiles exist in plasma from obese subjects [17], suggesting that some of these factors may modulate the systemic metabolic response. In this study, we aimed to evaluate the main effects of inflammation on the miRNA profile of differentiated human adipocytes, the macrophage-like cell line THP-1 (M1), and in their supernatants (SNs). Then, the miRNAs that experienced the most dramatic changes were evaluated in human subcutaneous AT before and after bariatric surgery-induced weight loss and the subsequent metabolic improvement and decreased inflammation.

## Results

### Comprehensive miRNA profiling in macrophages and adipocytes

**Figure 1:**
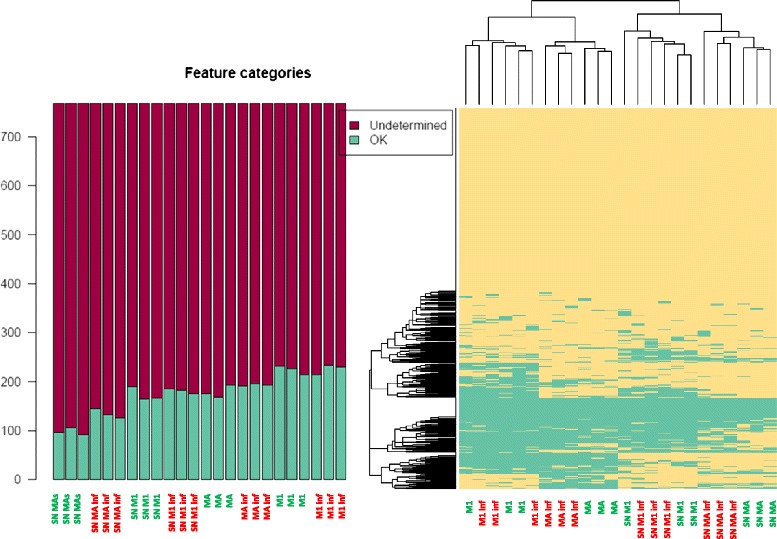
MicroRNA profiles and cluster analysis. MicroRNA profiles in cells and their supernatants (SN) after removing Ct values ≥35, and cluster analysis according to the quality of all determinations. Green (controls) for differentiated adipocytes (MAs) and M1 macrophages (M1) cultured under normal conditions. Inflamed (Inf) cells are shown in red.

**Figure 2:**
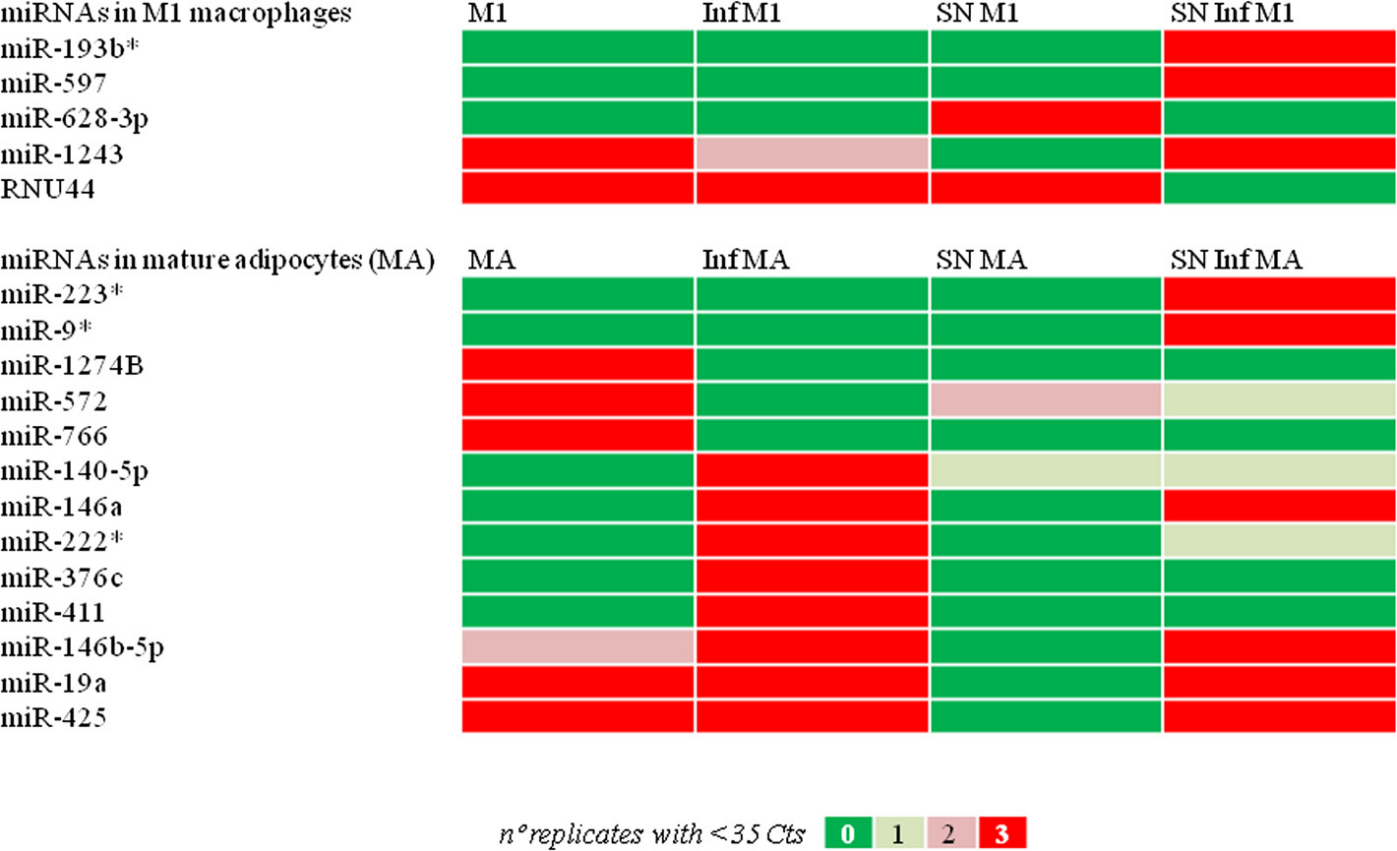
Major variations in miRNA profiles. Major variations in miRNA detection in cells and supernatants (SN) upon inflammation (Inf).

We measured the expression (cells) and the presence into the supernatant (SN) of 754 common mature miRNAs in differentiated human subcutaneous adipocytes and the macrophage-like THP-1 cell line (M1) at the baseline and upon inflammation (Figure 1). Differentiated adipocytes expressed 169 miRNAs (22.3% of the tested mature miRNAs), and 85 (11.2%) of these factors were found in the SN (Figure 1 and Additional file 1). Interestingly, changes regarding miRNA expression patterns and their concentration in SN clearly identified the inflammatory effects in isolated mature adipocytes cultured and differentiated *in vitro* (Figure 1). Indeed, some miRNAs became undetectable (miR-1274B, miR-572, and miR-766), while others appeared *de novo* in cells (miR-140-5p, miR-222*, miR-376c, miR-411, and miR-146a) and their SNs (miR-146a, miR-146b, miR-19a, miR-223*, miR-425, and miR-9*) upon MCM-induced inflammation (Figure 2). Overall, treatment with MCM increased the number of miRNAs expressed in adipocytes (+8.3%) and in their SN (+24.7%), concomitantly with increased *IL-6* (79-fold change, *P* = 0.004) and *TNFα* (14-fold change, *P* < 0.0001) gene expression (Table 1).

**Table 1:** **Significant modulation of miRNAs expressed and/or secreted (supernatant) by M1 macrophages upon lipopolysaccharide (LPS, 10 ng/ml) stimuli and mature adipocytes treated or not with macrophages LPS-conditioned medium (MCM, 5%)**

	**Expression**		**Supernatant**	
	**Ratio**	***P*** **value**	**Ratio**	***P*** **value**
	Inflamed (5% MCM)/control mature adipocytes (MAs)			
IL-6 mRNA	78.56	*0.004*		
TNFα mRNA	14	*<0.0001*		
miR-155	5.01	*0.015*	4.03	*<0.0001*
miR-132	3.17	*0.011*		
miR-222	2.51	*0.044*	6.07	*0.001*
miR-221	1.98	*0.027*	15.43	*0.002*
miR-17	1.60	*0.031*		
miR-31	1.59	*0.028*		
miR-93	1.59	*0.031*		
miR-345	1.58	*0.033*		
miR-422a	0.50	*0.028*		
miR-19b			19.76	*<0.0001*
miR-92a			7.73	*0.002*
miR-342-3p			6.08	*0.027*
miR-223			4.87	*0.001*
miR-20a			4.86	*0.001*
miR-199a-3p			0.47	*0.029*
miR-214			0.39	*0.026*
miR-193b			0.37	*0.014*
miR-152			0.24	*0.023*
	Inflamed (10 ng/ml LPS)/control macrophages (M1)			
IL-6 mRNA	17.74	*0.002*		
TNFα mRNA	4.90	*0.001*		
miR-146a	4.96	*<0.0001*		
miR-155	2.48	*0.001*		
miR-149	0.62	*0.012*		
miR-27a	0.61	*0.009*		
miR-19b	0.61	*0.009*		
miR-124	0.61	*0.011*		
miR-221	0.49	*0.034*		
miR-19a	0.49	*0.032*	2.02	*0.034*
miR-425*	0.41	*0.011*		
miR-181a	0.37	*0.015*		
miR-145	0.19	*0.005*	0.14	*0.032*
miR-378			4.95	*0.014*
miR-345			3.24	*0.013*
miR-132			2.92	*0.021*
miR-212			2.56	*0.013*
miR-106a			2.03	*0.035*
miR-25			1.93	*0.016*
miR-24			1.77	*0.005*
miR-92a			1.75	*0.007*
miR-30a-5p			1.55	*0.010*
miR-30e-3p			0.39	*0.002*
RNU48			0.22	*0.015*
miR-15b			0.20	*0.037*
let-7a			0.12	*0.014*

Results assessed were compared by Student *t*-test. Italics for significant results.

On the other hand, expression of 183 miRNAs (24%) and the presence in the SN of 106 (14%) were detected in M1 macrophages. Inflammatory stimuli with LPS increased the number of miRNAs expressed (+1.4% with respect to basal) and found in the SN (+5%) of differentiated M1 macrophages. Among them, miR-628-3p and RNU44 (detectable in SN at the basal level but not after treatment with LPS) and miR-1243, miR-193b*, and miR-597 (detectable in SN upon inflammation, Figure 2) changed their detection levels after stimulation. However, these qualitative changes did not reach significance enough to discriminate activated cells from controls (Figure 1). Indeed, the inflammatory reaction of LPS-stimulated M1 macrophages, as inferred from changes in the expression of well-recognized pro-inflammatory genes such as IL-6 and TNFα (17.7-fold change, *P* = 0.002, and 4.9-fold change, *P* = 0.001, respectively), did not show such great impact, when compared to the changes accounted in mature adipocytes upon inflammation (Table 1). The lack of major differences in stimulated M1 macrophages was more probably due to the transient basal inflammatory state that the induction of the mature type 1 macrophage-like state M1 (by treating THP-1 cells with phorbol 12-myristate 13-acetate) promotes *per se*. Nonetheless, under basal conditions adipocytes and M1 macrophages shared expression of 135, and 71 common mature miRNAs were found in SNs from both cell lines. Upon inflammatory stimuli, differentiated adipocytes and isolated M1 macrophages expressed 147 (+9%) common mature miRNA species, and 100 (+41%) of these factors were found in the SN (Additional file 2: Table S1).

### Quantitative changes upon inflammation

**Figure 3:**
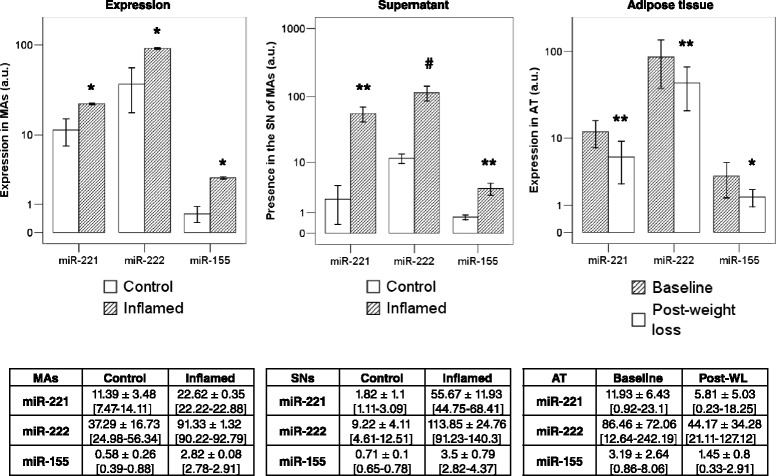
Bars representing the mean ± standard error for miR-221, miR-222, and miR-155. Bars representing the mean ± standard error for miR-221, miR-222, and miR-155 in differentiated adipocytes and their SN at the baseline and upon MCM-conditioned inflammation (striped bars) and in human AT before (striped bars)-after massive weight loss. The geometric average of all analyzed miRNAs was used as normalizing factor in cells and the SNs, respectively. RNU6b was used as reference miRNA in human adipose tissue. Bottom tables provide normalized values as mean ± standard deviation (minimum-maximum). *P < 0.05, **P < 0.01, and ^#^ *P* < 0.0001 (Student’s or paired *t*-test) for comparisons treatment *vs*. control, pre- *vs*. post-weight loss.

When comparing normalized values of quantifiable miRNAs (Cts <35 in all replicates) either in cells or the SNs of M1 macrophages and isolated adipocytes following or not treatment (inflammation vs. control), significant changes in 18 mature miRNAs were identified in mature adipocytes and 24 in M1 macrophages (Table 1). Among them, increased expression of miR-221 (2-fold, *P* = 0.002), miR-222 (2.5-fold, *P* = 0.04), and miR-155 (5-fold, *P* = 0.015, Figure 3) was found in inflamed adipocytes, as well as in the SN (15-, 6-, and 4-fold changes, respectively, all with *P* values under 0.001, Figure 3), when compared to control. Other significant changes in isolated adipocytes included increased expression of miR-132, miR-17, miR-31, miR-93, and miR-345 and decreased miR-422a expression. In the SN, increased concentrations of miR-19b, miR-92a, miR-342-3p, miR-223, and miR-20a and decreased miR-199a-3p, miR-214, miR-193b, and miR-152 were also identified (Table 1).

On the other hand, increased expressions of miR-155 (2.5-fold, *P* = 0.001) but decreased miR-221 (0.49-fold, *P* = 0.034) were found in LPS-stimulated M1 macrophages (Table 1). Although no significant variations were identified in miR-222, modulation of miR-19a (0.5-fold, *P* = 0.032, and 2-fold, *P* = 0.034) and decreased miR-145 in both cells and SNs (0.2-fold, *P* = 0.005, and 0.14-fold, *P* = 0.032) were shown in M1 macrophages reacting to LPS (Table 2). Other relevant changes in macrophages included increased miR-146a (5-fold change, *P* < 0.0001), and the decreased expression of nine specific mature miRNAs, which were superseded by increased presence in the SN of another set of nine different miRNAs (Table 1).

**Table 2:** **Testing of inflammation-induced miRNA candidates identified** ***in vitro*** **in human subcutaneous adipose tissue (AT) samples**

	**Inflamed (5% MCM)/control adipocytes**				**Inflamed (10 ng/ml LPS)/control macrophages**				**Obese AT/post-weight loss**	
	**Expression**		**Supernatant**		**Expression**		**Supernatant**		**Human AT (** ***n*** **= 9)**	
	**Ratio**	***P*** **value**	**Ratio**	***P*** **value**	**Ratio**	***P*** **value**	**Ratio**	***P*** **value**	**% change**	***P*** **value**
miR-146b	Only detectable in the SN of inflamed MAs								−88	*<0.0001*
miR-146a					4.96	*<0.0001*			−69.6	*0.002*
miR-376c	Only detectable in inflamed MAs								−78	*0.002*
miR-221	1.98	*0.027*	15.43	*0.002*	0.49	*0.034*			−51.3	*0.003*
miR-223			4.87	*0.001*					−74.3	*0.004*
miR-411	Only detectable in inflamed MAs								−62.2	*0.004*
miR-155	5.01	*0.015*	4.03	*<0.0001*	2.48	*0.001*			−54.4	*0.005*
miR-19b			19.76	*<0.0001*	0.61	*0.009*			−72.7	*0.006*
miR-19a	Only detectable in the SN of inflamed MAs				0.49	*0.032*	2.02	*0.034*	−73.4	*0.008*
RNU48							0.22	*0.015*	−45	*0.009*
miR-222	2.51	*0.044*	6.07	*0.001*					−48.9	*0.028*
miR-193b			0.37	*0.014*					−44.8	*0.039*
miR-766	Undetectable in inflamed MAs								182	0.084
let-7a							0.12	*0.014*	6.1	0.276
miR-145					0.19	*0.005*	0.14	*0.032*	−26.9	0.228
miR-92a			7.73	*0.002*			1.75	*0.007*	−21.3	0.327
miR-572	Undetectable in inflamed MAs								−7.6	0.413
miR-378							4.95	*0.014*	−41	0.78

Results from nine morbid obese participants before and after surgery-induced weight loss (post-weight loss, see Additional file 2: Table S2 for additional details). Results assessed were compared by paired *t*-test. ‘Detectable’ refers to <35 Cts, while ‘undetectable’ indicates ≥35 Cts in all biological replicates (*n* = 3), as explained above. Italics for significant results.

### *In vivo* validation

Noteworthy, qRT-PCR analyses in AT from morbid obese subjects following approximately 2 years of weight loss identified significant changes in 12 of 18 miRNAs (67%) shortlisted by analyses *in vitro* (Table 2). Currently, it is well recognized that, when compared with conventional strategies, the physiological impact of bariatric surgery is of particular interest, with regard to short-term weight loss and improvement of inflammation [18]. Indeed, concomitantly with weight loss and improved AT inflammation (Additional file 2: Table S2), current data pointed decreased AT expression of miRNAs which detection inside isolated cells (for example, miR-146b, miR-376c, miR-411) and/or in the SN (miR-221, miR-222, miR-155, miR-223, miR-19a/b) rose in differentiated adipocytes upon inflammatory stimuli (Table 2). On the other hand, some miRNAs highlighted by either differential expression or concentrations in the SN of inflamed M1 macrophages, such as miR-146a, miR-155, and miR-19a, also showed decreased expression in human obese AT after weight loss (Table 2).

## Discussion

Current results indicate that inflammation alters miRNA profiles in adipocytes and macrophages. Adipose tissue (AT) exhibits inflammation as tissue mass expands in obesity, involving macrophage infiltration and the inflammatory response of adipocytes [8]. Analyses of AT before and after weight loss have revealed a reduction in macrophage number and decreased expression of pro-inflammatory genes [7]. There is preliminary information about the potential involvement of post-transcriptional regulatory elements such as microRNAs (miRNAs) in this context [19]. Indeed, secreted miRNAs may target neighboring cells and perform regulatory functions in them [3,19,20].

The most recent studies have revealed that plasma membrane-derived vesicles, namely exosomes and microvesicles, can transfer miRNAs from donor to recipient cells [21]. These extracellular vesicles are not simply ‘garbage bags’ or a route for exclusion of surplus molecules but active effectors in mediating adaptive responses in targeting cells [22]. Others have demonstrated that more extracellular vesicles containing miRNAs are released in *ex vivo* adipose tissue cultures of mice feeding a high-fat diet and of leptin-deficient obese mice when compared to control mice [23]. These exosomes released to the supernatant are predicted to have both local and systemic effects, being taken up by resident macrophages and leading to their activation in adipose tissue. Because of this activation, more macrophages may be recruited into AT to further increase the inflammatory response, with more miRNAs released from inflamed AT [23]. Also, HDL transports large amounts of endogenous miRNAs, being suggested as the possible mechanism whereby some of the biological effects of HDL could be explained [3]. The molecular mechanisms of RNA-based cell-to-cell communication and the exact process of how extracellular vesicles and HDL are loaded with miRNAs have not been characterized yet and, thus, the pathways involved remain largely unknown. However, since significant differences regarding miRNAs contained in AT [14] and the circulation [17] of obese patients exist, expression and secretion by inflamed adipocytes and activated M1 macrophages might be attractive candidates in this field.

### Inflammation alters miRNA profiles in adipocytes and macrophages

Upon inflammation, an increased number of miRNAs were detectable in stimulated adipocytes and M1 macrophages, when compared to the basal conditions. This increase was more noticeable when studying the miRNAs that are found in the supernatant (SN) of adipocytes. Identification of miR-146a and miR-146b in adipocytes upon inflammation (but not under basal conditions), for example, was consistent when analyzing both cells and SNs, decreasing in human AT after weight loss. This agrees with the notion that AT miR-146 increases as fat mass expands in mice models of obesity [24]. On the other hand, the expression of ‘miRNokines’ such as miR-146b, miR-376c, miR-411, and miR-19a by inflamed adipocytes/obese AT may hint into functional activities beyond adiposity, such as recruitment of immune cells [25], or modulation of insulin-secreting cells [26]. For instance, miR-19b directly regulates the formation of ‘foam cells’ derived from macrophages [27] and was measurable here in the SN of LPS-activated M1 macrophages (but not in controls). This may suggest mechanisms by which cells release a particular set of miRNAs to the SN and miRNAs which are more likely to be kept inside cells. Further investigations are needed in order to determine these mechanisms.

In differentiated adipocytes, miR-221, miR-222, and miR-155 were detectable and measurable in both cells and SNs, being their values significantly increased upon inflammation. These three miRNA candidates were also associated with obesity and weight loss, when analyzed in AT samples. Indeed, this increased expression in obese AT has been reported previously [24,14], as well as their association with AT oxidative stress, apoptosis, and inflammatory events [28]. In agreement with current results, macrophages in atherosclerotic lesions exhibit increased expression of miR-155, which also responds to inflammatory cues [29]. On the other hand, TNFα induced the expression of both miR-221 and miR-222, which were also found to be downregulated during adipogenesis and upregulated in obese mice [30]. The concordant and sustained changes in the expression of these three miRNAs seem to be connected with inflammatory nodes in obese AT, suggesting their participation in regulatory networks underlying adipogenesis and AT dysfunction. In this respect, three major target genes [c-Cbl protein, phosphoinositide-3-kinase regulatory subunit 1 (*PI3KR1*), and the suppressor of cytokine signaling 1 (*SOCS1*)] for the *in silico* intersection between two of the more relevant miRNAs in the context of inflammation affecting fat cells, the miR-155 and miR-221 (Additional file 1: Table S3), are involved in insulin pathway (Additional file 3: Figure S2) and, thus, may participate of insulin resistance and type 2 diabetes (Additional file 3: Figure S3) [31]. The *PIK3R1* gene, encoding the p85 regulatory subunit of phosphatidylinositol 3-kinase (PI3K), couples insulin and leptin signaling pathways, playing a critical role in mediating AT insulin sensitivity, and the initiation and propagation of the inflammatory response [32]. Also, the proto-oncogene Casitas b-lineage lymphoma (CBL) plays an important role in AT hyperplasia and insulin action, independently of the PI3K/Akt pathway [33]. The ability of insulin to stimulate glucose uptake relies on a complex signaling cascade that leads to the translocation of glucose receptors to the membrane. Thus, the modulation of expression and secretion of these miRNAs in AT, particularly in adipocytes, may contribute to the improvement of insulin sensitivity following massive fat mass loss and decreased inflammation. Understanding the miRNA profiles associated with different pathways for macrophage activation could also provide a new spectrum of biomarkers for identification and a potential therapeutic approach to mitigate entry into tissues and the onset of inflammatory diseases.

### miRNA profiles in macrophages and adipocytes and their SNs

Type 1 macrophage-like human monocytes and human subcutaneous differentiated adipocytes share expression of several miRNAs, being this expression enhanced in the former. The data agrees with accumulating evidence that adipocytes and immune cells show common features, with expression of genes previously considered to be specific of one lineage [34], and the concept that pro-inflammatory components are not only secreted by the stromal-vascular cell fraction but also by differentiated adipocytes [35]. Notably, when comparing inflamed adipocytes and M1 macrophages, the number of miRNAs that were only detectable in adipocytes or in macrophages decreased, especially when analyzing their SNs. Thus, the similitude between mature adipocytes and macrophages [36] becomes clearer through concomitant changes in miRNA expression pattern and the miRNAs found in the SN when inflammation appears.

## Conclusions

Current results highlight the existence of cell-dependent miRNA profiles inside and in the SN of isolated adipocytes and M1 macrophages. Obesity-related inflammation increases the production of miRNAs and alters the expression pattern in both cells and SNs. Understanding miRNA profiles associated with different pathways of adipocytes and macrophages activation may provide a new spectrum of biomarkers and therapeutic approaches to mitigate macrophage infiltration in obese AT and the onset of obesity-associated inflammation. Variations regarding miR-221/222 and miR-155, for example, may participate in the crosstalk between obesity-related inflammation, insulin resistance, and other obesity-associated morbidities. This work paves the way for future clinical and cellular studies aimed at determining the impact of these molecular adaptations on the development of obesity-related inflammation and such deleterious effects.

## Methods

### Cell culture

The human monocyte cell line THP-1 (ATCC®) was cultured in RPMI 1640 medium containing 10% fetal bovine serum, 5 mM glucose, 2 mM L-glutamine, 50 mg/ml gentamicin, and 20 mM HEPES at 37°C in a humidified 5% CO_2_ and 95°C air atmosphere. The mature type 1 macrophage-like state (M1) was induced by treating THP-1 cells with 0.162 mM of phorbol 12-myristate 13-acetate (PMA) for 24 h. Differentiated, plastic-adherent cells were washed with cold Dulbecco’s phosphate-buffered saline (Sigma Chemical Co., St. Louis, MO, USA) and incubated with fresh medium without PMA. Differentiated M1 macrophages were treated with fresh medium and fresh medium containing 10 ng/ml of lipopolysaccharides (LPS, Sigma Chemical Co.) for an additional 24-h period (three biological replicates per treatment), as previously described [5]. Then, the medium was collected and centrifuged at 400 · *g* for 5 min, diluted with adipocyte medium (Zen-Bio, Inc., Research Triangle Park, NC, USA), and used as macrophage LPS-conditioned media (MCM, 5%) to induce inflammation in differentiated human adipocytes (MAs).

Human subcutaneous preadipocytes from a non-diabetic Caucasian male with body mass index (BMI) <30 and age <40 (Zen-Bio, Inc.) were cultured with the Preadipocytes Medium (PM, Zen-Bio, Inc.) in a humidified 37°C incubator with 5% CO_2_ and 95°C air atmosphere. Twenty-four hours after plating, cells were checked for confluence and differentiated using the commercially available Differentiation Medium (DM, Zen-Bio, Inc.) following manufacturer’s instructions. Two weeks after initializing differentiation, cells appeared rounded with large lipid droplets in the cytoplasm. Cells were considered mature adipocytes and incubated with fresh adipocytes media (control) or fresh media containing 5% MCM, to mimic the inflammatory milieu of obese AT. After 24 h of treatment, the supernatants were centrifuged at 400 · *g* for 5 min, and both pellets and supernatants were removed and stored at −80°C for future analysis. Three biological replicates per treatment were performed and analyzed (Additional file 3: Figure S1 - Workflow diagram).

### Subject recruitment

Expression and validation of the miRNAs shortlisted *in vitro* was assessed in subcutaneous abdominal adipose tissue (AT) from nine morbidly obese women before and after approximately 2 years of surgery-induced weight loss (BMI = 43.4 ± 5 kg/m^2^, age = 48 ± 10 years [mean ± SD], Additional file 2: Table S2). These volunteers were recruited at the Department of Diabetes, Endocrinology and Nutrition and the Department of Surgery of the Hospital ‘Dr Josep Trueta’ of Girona (Girona, Spain). All subjects provided written informed consent before entering the study, were of Caucasian origin, and reported that their body weight was stable for at least 3 months before entering the study. No systemic diseases other than obesity were reported. All participants were free of any infections in the previous month before entering the study. Liver and thyroid dysfunction were specifically excluded by biochemical work-up. Other exclusion criteria included the following: 1) clinically significant hepatic, neurological, or other major systemic disease, including malignancy, 2) history of drug or alcohol abuse or serum transaminase activity more than twice the upper limit of normal, 3) an elevated serum creatinine concentration, 4) acute major cardiovascular event in the previous 6 months, 5) acute illnesses and current evidence of high-grade chronic inflammatory or infective diseases, and 6) mental illness rendering the subjects unable to understand the nature, scope, and possible consequences of this study. The study protocol was approved by the Ethics Committee and the Committee for Clinical investigation (CEIC) of the Hospital ‘Dr. Josep Trueta’ of Girona, so we certify that all applicable institutional regulations concerning the ethical use of information and samples from human subjects were followed during this research.

### RNA extraction

Total RNA, including small RNA species such as miRNAs, was extracted and purified from AT and cells (macrophages and adipocytes) using miRNeasy® Mini Kit (QIAGEN, Gaithersburg, MD, USA). AT samples (approximately 150 μg) and cells were homogenized in 0.6 mL of QIAzol® Lysis Reagent (QIAGEN), a monophasic solution of phenol and guanidine thiocyanate which facilitates sample disaggregation and inhibits RNAses. After addition of chloroform (0.4 volumes), the homogenate is separated into aqueous and organic phases by centrifugation (15 min at 12,000 · *g* and 4°C). Then, the upper aqueous RNA-rich phase was isolated and ethanol absolute (1.5 volumes) was added to provide appropriate binding conditions for all RNA molecules, including miRNAs. The sample was applied to a silica-membrane RNeasy spin columns, where RNA binds to the membrane while phenols and other compounds are washed away. High-quality RNA was finally eluted in 30 μL of RNAse-free water. Final RNA concentrations were assessed with a Nanodrop ND-1000 Spectrophotometer (Thermo Fischer Scientific, Wilmington, DE, USA). The integrity was checked with the Nano lab-on-a-chip assay for total eukaryotic RNA using Bioanalyzer 2100 (Agilent Technologies, Palo Alto, CA, USA).

miRNA profiles were assessed in cell supernatant (SN) following standard procedures previously optimized for plasma [17]. First, floating cells were removed from the media by an additional centrifugation of 2,000 · *g* for 5 min at 4°C using a laboratory centrifuge (Beckman J-6M Induction Drive Centrifuge, Palo Alto, CA, USA). RNA extraction from cell media was performed using the mirVana PARIS Isolation Kit (Applied Biosystems, Darmstadt, Germany), as previously described [17].

### Retrotranscription to cDNA and pre-amplification

A 2,000 ng of total RNA from AT and cell debris and a fixed volume of 3 μL of RNA solution from the 40-μL eluate of RNA isolation performed in SN were used as input into the reverse transcription (RT), using the TaqMan miRNA Reverse Transcription Kit and TaqMan miRNA Multiplex RT Assays, which are required to run the TaqMan Array microRNA Cards (Applied Biosystems). Pre-amplification was performed in RNA samples from SN using TaqMan PreAmp Master Mix and Megaplex™ PreAmp Primers (human pool set A and B), which provided an optional amplification step prior to real-time analysis when the RNA sample is limiting. This step is mandatory to provide reliable results in the SN, as well as in plasma [17].

### miRNA profiling using TLDAs

TaqMan low-density arrays (TLDAs) for miRNAs, covering a total amount of 754 miRNA species, were applied to three biological replicates. Real-time (RT)-PCR was carried out on an Applied Biosystems 7900HT thermocycler. Data were analyzed with SDS Relative Quantification Software version 2.2.2 (Applied Biosystems), with an assigned minimum threshold above the baseline of all assays showing measurable amplifications above background and threshold thermal cycles (Cts) below 35. The geometric average of all analyzed miRNAs was used as normalizing factor in cells and the SN. Normalized relative log_10_ ratios were used for posterior statistical tests, as previously described [37].

### Analysis of individual miRNAs using TaqMan hydrolysis probes

Commercially available TaqMan hydrolysis probes (Applied Biosystems) were used to assess the expression of individual miRNAs in human AT. After RNA extraction and retrotranscription of 2,000 ng of RNA, the product was diluted (1:100) previous being combined (5 μL) with 0.25 μL of TaqMan miRNA hydrolysis probes (20×) and 4.75 μL of the LightCycler 480 Probes master mix (2×) (Roche Diagnostics, Barcelona, Spain) to a final volume of 10 μL. Gene expression was assessed by real-time PCR using the LightCycler® 480 Real-Time PCR System (Roche Diagnostics, Barcelona, Spain) and TaqMan technology suitable for relative gene expression quantification following manufacturer’s protocol. RNU6b (NCBI Accession ID#: NR_002752, assay ID#: 4395470) was used as reference miRNA (or endogenous control) in human AT.

### Gene expression

Three micrograms of total RNA were reverse transcribed to cDNA using High-Capacity cDNA® Archive Kit (Applied Biosystems, Darmstadt, Germany) according to manufacturers’ protocols. Commercially available and pre-validated TaqMan® primer/probe sets were used for gene expression determinations (Applied Biosystems). Expression was assessed by real-time PCR using the LightCycler® 480 Real-Time PCR System (Roche Diagnostics, Barcelona, Spain) and TaqMan® technology suitable for relative gene expression quantification. The reaction was performed following manufacturers’ instructions in a final volume of 7 μl. The cycle program consisted of an initial denaturing of 10 min at 95°C then 45 cycles of 15-s denaturizing phase at 92°C and 1-min annealing and extension phase at 60°C. Then, the crossing point (Cp) values were assessed for each amplification curve by the second derivative maximum method. ∆Cp value was first calculated by subtracting the Cp value for corresponding endogenous controls in each sample from the Cp value for each sample and target gene. Fold changes compared with the endogenous control were then determined by calculating 2^−∆Cp^, so gene expression results are expressed as expression ratio relative to preselected and validated endogenous control. The commercially available and pre-validated TaqMan® primer/probe sets used here for gene expression were as follows: interleukin 6 (IL-6, Hs00985639_m1), tumor necrosis factor (TNFα, Hs01113624_g1), and leptin (LEP, Hs00174877_m1). Peptidyl-prolyl cis-trans isomerase A (PPIA, Hs99999904_m1, also known as cyclophilin A) expressions were assessed as endogenous control in each reaction and sample. Replicates and positive and negative controls were included.

### Statistical methods

Tables were constructed for qualitative evaluation of the number and identity of miRNAs shared among all samples and cells. Tables for each group of treatments indicate the number of observed raw RT-PCR values below 35 Cts in three biological replicates. The detectability of miRNA in cells or SN under different conditions was established on less than 35 Cts for all replicates (*n* = 3). Regarding quantitative differences, normal distribution and homogeneity of variances were evaluated using Levene’s test before conducting statistical analysis. Variables were given in a base log_10_ transformation and analyzed on that log_10_ scale when necessary. Student’s or paired *t*-tests were performed to study differences between groups of samples. Data analyses were performed with the SPSS statistical software (SPSS v13.0, IBM, Chicago, IL, USA) and the *R* Statistical Software (http://www.r-project.org/). Potential miRNA target genes and pathways were identified using DIANA-mirPath [31] and TargetScan 6.0 [38].

## Additional files

Additional file 1:
**Supplemental information - raw data.**
Additional file 2: Table S1. Most significant differences regarding miRNA availability in macrophages (M1) and adipocytes (MAs) and into supernatants (SNs) at the basal level and upon inflammation (Inf). **Table S2.** Anthropometric and biochemical data and expression results in subcutaneous adipose tissue samples from nine participants recruited for validation *in vivo* before (baseline) and after (post-weight loss) bariatric surgery. **Table S3.** Intersection of *in silico* identified target genes of miR-155 and miR-221. ^†^Their involvement in insulin signaling is represented in Additional file 3: Figure S2 and S3. Additional file 3: Figure S1. Workflow diagram. **Figure S2.** Insulin signaling pathway showing the involvement of CBL, SOCS1, and PIK3R1 (yellow boxes). Their post-transcriptional regulation may be accomplished by both miR-221 and miR-155. **Figure S3.** SOCS1 and PIK3R1 (yellow boxes) in the context of type II diabetes and impaired glucose intake.
