# Supplementary material for: Inflammation triggers specific microRNA profiles in human adipocytes and macrophages and in their supernatants
Source: Clin Epigenetics. 2015 Apr 24;7(1):49. doi: 10.1186/s13148-015-0083-3 (PMC4413548; doi:10.1186/s13148-015-0083-3)
Supplement: Additional file 2: Table S1. — Most significant differences regarding miRNA availability in macrophages (M1) and adipocytes (MAs) and into supernatants (SNs) at the basal level and upon inflammation (Inf). Table S2. Anthropometric and biochemical data and expression results in subcutaneous adipose tissue samples from nine participants recruited for validation in vivo before (baseline) and after (post-weight loss) bariatric surgery. Table S3. Intersection of in silico identified target genes of miR-155 and miR-221. †Their involvement in insulin signaling is represented in Additional file 3: Figure S2 and S3. [file 13148_2015_83_MOESM2_ESM.doc]

**Table S1** Most significant differences regarding miRNAs availability in macrophages (M1) and adipocytes (MAs) and into supernatants (SN) at the basal level and upon inflammation (Inf).

| **miRNA-assay ID#** | **MAs** | **M1** |  | **miRNA-assay ID#** | **Inf MAs** | **Inf M1** |
| --- | --- | --- | --- | --- | --- | --- |
| miR-127-3p-4373147 |  |  |  | miR-145*-002149 |  |  |
| miR-138-4395395 |  |  |  | miR-206-000510 |  |  |
| miR-193b*-002366 |  |  |  | miR-214*-002293 |  |  |
| miR-206-000510 |  |  |  | miR-214-4395417 |  |  |
| miR-214*-002293 |  |  |  | miR-22-4373079 |  |  |
| miR-296-5p-4373066 |  |  |  | miR-299-5p-4373188 |  |  |
| miR-31*-002113 |  |  |  | miR-31*-002113 |  |  |
| miR-31-4395390 |  |  |  | miR-31-4395390 |  |  |
| miR-409-3p-002332 |  |  |  | miR-376c-4395233 |  |  |
| miR-433-4373205 |  |  |  | miR-409-3p-002332 |  |  |
| miR-495-4381078 |  |  |  | miR-411-4381013 |  |  |
| miR-539-4378103 |  |  |  | miR-485-3p-4378095 |  |  |
| miR-572-001614 |  |  |  | miR-495-4381078 |  |  |
| miR-615-3p-4386777 |  |  |  | miR-539-4378103 |  |  |
| miR-106b*-002380 |  |  |  | miR-106b*-002380 |  |  |
| miR-130b*-002114 |  |  |  | miR-126*-000451 |  |  |
| miR-130b-4373144 |  |  |  | miR-142-3p-4373136 |  |  |
| miR-140-5p-4373374 |  |  |  | miR-15a*-002419 |  |  |
| miR-142-3p-4373136 |  |  |  | miR-15b*-002173 |  |  |
| miR-146a-4373132 |  |  |  | miR-185-4395382 |  |  |
| miR-148b-4373129 |  |  |  | miR-18a*-002423 |  |  |
| miR-151-5P-002642 |  |  |  | miR-18a-4395533 |  |  |
| miR-15a*-002419 |  |  |  | miR-200b-4395362 |  |  |
| miR-15b*-002173 |  |  |  | miR-200c-4395411 |  |  |
| miR-18a*-002423 |  |  |  | miR-20a*-002437 |  |  |
| miR-18a-4395533 |  |  |  | miR-20b-4373263 |  |  |
| miR-18b-4395328 |  |  |  | miR-223*-002098 |  |  |
| miR-200c-4395411 |  |  |  | miR-223-4395406 |  |  |
| miR-20a*-002437 |  |  |  | miR-340-4395369 |  |  |
| miR-20b-4373263 |  |  |  | miR-425*-002302 |  |  |
| miR-220-4373078 |  |  |  | miR-483-5p-4395449 |  |  |
| miR-222*-002097 |  |  |  | miR-505-4395200 |  |  |
| miR-223*-002098 |  |  |  | miR-550-001544 |  |  |
| miR-30d*-002305 |  |  |  | miR-582-3p-4395510 |  |  |
| miR-340*-002259 |  |  |  | miR-590-5p-4395176 |  |  |
| miR-340-4395369 |  |  |  | miR-598-4395179 |  |  |
| miR-424-4373201 |  |  |  | miR-625-4395542 |  |  |
| miR-425*-002302 |  |  |  | miR-629-001562 |  |  |
| miR-483-5p-4395449 |  |  |  | miR-766-001986 |  |  |
| miR-500-4395539 |  |  |  | miR-885-5p-4395407 |  |  |
| miR-503-4373228 |  |  |  | miR-9*-002231 |  |  |
| miR-517b-4373244 |  |  |  | miR-92a-1*-002137 |  |  |
| miR-550-001544 |  |  |  | miR-941-002183 |  |  |
| miR-590-5p-4395176 |  |  |  | miR-942-002187 |  |  |
| miR-598-4395179 |  |  |  | miR-9-4373285 |  |  |
| miR-629-001562 |  |  |  |  |  |  |
| miR-885-5p-4395407 |  |  |  |  |  |  |
| miR-9*-002231 |  |  |  |  |  |  |
| miR-92a-1*-002137 |  |  |  |  |  |  |
| miR-9-4373285 |  |  |  |  |  |  |
| rno-miR-7*-001338 |  |  |  |  |  |  |

| nº replicates with <35 Cts | **0** | **3** |
| --- | --- | --- |

| **miRNA-assay ID#** | **SN MAs** | **SN M1** |  | **miRNA-assay ID#** | **SN Inf MAs** | **SN Inf M1** |
| --- | --- | --- | --- | --- | --- | --- |
| miR-10b*-002315 |  |  |  | miR-31-4395390 |  |  |
| miR-125b-4373148 |  |  |  | miR-101-4395364 |  |  |
| miR-206-000510 |  |  |  | miR-139-5p-4395400 |  |  |
| dme-miR-7-000268 |  |  |  | miR-15a*-002419 |  |  |
| let-7d-4395394 |  |  |  | miR-18a*-002423 |  |  |
| let-7g-4395393 |  |  |  | miR-193b*-002366 |  |  |
| miR-103-4373158 |  |  |  | miR-26a-4395166 |  |  |
| miR-122-4395356 |  |  |  | miR-27a*-002445 |  |  |
| miR-1290-002863 |  |  |  | miR-29a*-002447 |  |  |
| miR-130b-4373144 |  |  |  | miR-324-5p-4373052 |  |  |
| miR-146a-4373132 |  |  |  | miR-483-5p-4395449 |  |  |
| miR-146b-5p-4373178 |  |  |  | miR-577-002675 |  |  |
| miR-15b-4373122 |  |  |  | miR-590-5p-4395176 |  |  |
| miR-181a-4373117 |  |  |  | miR-597-4380960 |  |  |
| miR-185-4395382 |  |  |  | miR-766-001986 |  |  |
| miR-18a*-002423 |  |  |  | miR-874-4395379 |  |  |
| miR-18b-4395328 |  |  |  |  |  |  |
| miR-19a-4373099 |  |  |  |  |  |  |
| miR-210-4373089 |  |  |  |  |  |  |
| miR-211-4373088 |  |  |  |  |  |  |
| miR-212-4373087 |  |  |  |  |  |  |
| miR-222*-002097 |  |  |  |  |  |  |
| miR-223*-002098 |  |  |  |  |  |  |
| miR-23a-4373074 |  |  |  |  |  |  |
| miR-26b-4395167 |  |  |  |  |  |  |
| miR-28-5p-4373067 |  |  |  |  |  |  |
| miR-301a-4373064 |  |  |  |  |  |  |
| miR-324-5p-4373052 |  |  |  |  |  |  |
| miR-339-3p-4395295 |  |  |  |  |  |  |
| miR-340*-002259 |  |  |  |  |  |  |
| miR-34c-5p-4373036 |  |  |  |  |  |  |
| miR-378-000567 |  |  |  |  |  |  |
| miR-425*-002302 |  |  |  |  |  |  |
| miR-425-4380926 |  |  |  |  |  |  |
| miR-532-3p-4395466 |  |  |  |  |  |  |
| miR-577-002675 |  |  |  |  |  |  |
| miR-598-4395179 |  |  |  |  |  |  |
| miR-628-3p-002434 |  |  |  |  |  |  |
| miR-629-001562 |  |  |  |  |  |  |
| miR-744-4395435 |  |  |  |  |  |  |
| miR-766-001986 |  |  |  |  |  |  |
| miR-769-5p-001998 |  |  |  |  |  |  |
| miR-9*-002231 |  |  |  |  |  |  |
| miR-942-002187 |  |  |  |  |  |  |
| miR-9-4373285 |  |  |  |  |  |  |
| rno-miR-7*-001338 |  |  |  |  |  |  |
| RNU44-4373384 |  |  |  |  |  |  |

| nº replicates with <35 Cts | **0** | **3** |
| --- | --- | --- |

**Table S2** Anthropometric and biochemical data and expression results in subcutaneous adipose tissue samples from 9 participants recruited for validation *in vivo* before (baseline) and after (post-weight loss) bariatric surgery.

| Parameters | Longitudinal comparisons (n=9) | | |
| --- | --- | --- | --- |
| Clinical data | Baseline | Post-weight loss | p-value^a^ |
| Age (Yrs) | 48 ± 10 | 51 ± 9 | **<0.0001** |
| BMI (kg / m^2^) | 43.70 ± 5.04 | 31.98 ± 5.95 | **<0.0001** |
| Fat mass (%) | 54.02 ± 8.19 | 40.93 ± 8.45 | **<0.0001** |
| SBP (mmg) | 131.44 ± 18.24 | 133.78 ± 19.01 | 0.736 |
| DBP (mmg) | 78.89 ± 11.94 | 73.22 ± 9.13 | 0.192 |
| Glucose (mg / dL) | 93.44 ± 8.93 | 89.67 ± 5.32 | 0.24 |
| Cholesterol (mg / dL) | 175.11 ± 21.50 | 175.56 ± 39.67 | 0.965 |
| LDL (mg / dL) | 57.18 ± 11.05 | 71.11 ± 28.41 | 0.091 |
| HDL (mg / dL) | 98.11 ± 17.25 | 88.78 ± 22.33 | 0.145 |
| Triglycerides (mg / dL) | 99.33 ± 41.78 | 77.67 ± 26.11 | 0.138 |
| Leucocytes (K / mcl) | 7.96 ± 2.51 | 5.82 ± 1.48 | **0.03** |
| CRP (mg / dl) | 0.903 ± 0.705 | 0.068 ± 0.017 | **0.048** |
| IL-6 mRNA (a.u.) | 0.00325 ± 0.00662 | 0.00078 ± 0.00029 | 0.304 |
| TNFa mRNA (a.u.) | 0.00354 ± 0.00261 | 0.00099 ± 0.00049 | **0.013** |
| LEP mRNA (a.u.) | 0.977 ± 0.677 | 0.436 ± 0.188 | **0.031** |

Values represent the mean ± SD. BMI: body mass index, SBP: systolic blood pressure, DBP: diastolic blood pressure, HbA1C: glycated hemoglobin, HDL: high-density lipoprotein, LDL: low-density lipoprotein, IL-6: interlekin-6, TNFa: tumor necrosis factor alpha, LEP: leptin. ^a^ Results post-weight loss vs. baseline were compared by paired t-test. Bold indicates significant results (p-value < 0.05). a.u. arbitrary units.

**Table S3** Intersection of *in silico* identified target genes of miR-155 and miR-221. † Their involvement in insulin signaling is represented in **Figure S2** and S**3**.

| Intersection (miR-155 and miR-221) | | | | |
| --- | --- | --- | --- | --- |
| Total genes: 29 / Found in pathways: 10 | | | | |
| KEGG Pathway | Gene Name | -ln (p-value) | Ensembl Gene ID | Pathway ID |
| T cell receptor signaling pathway | FOS, CBL, PIK3R1, NFAT5 | 27.16 | ENSG00000170345 ENSG00000110395 ENSG00000145675 ENSG00000102908 | hsa04660 |
| B cell receptor signaling pathway | FOS, PIK3R1, NFAT5 | 21.08 | ENSG00000170345 ENSG00000145675 ENSG00000102908 | hsa04662 |
| Chronic myeloid leukemia | CBL, E2F2, PIK3R1 | 16.45 | ENSG00000110395 ENSG00000007968 ENSG00000145675 | hsa05220 |
| Type II diabetes mellitus † | SOCS1, PIK3R1 | 11.94 | ENSG00000185338 ENSG00000145675 | hsa04930 |
| Insulin signaling pathway † | SOCS1, CBL, PIK3R1 | 8.84 | ENSG00000185338 ENSG00000110395 ENSG00000145675 | hsa04910 |
| Non-small cell lung cancer | E2F2, PIK3R1 | 8.58 | ENSG00000007968 ENSG00000145675 | hsa05223 |
| Jak-STAT signaling pathway | SOCS1, CBL, PIK3R1 | 7.97 | ENSG00000185338 ENSG00000110395 ENSG00000145675 | hsa04630 |
| Glioma | E2F2, PIK3R1 | 7.32 | ENSG00000007968 ENSG00000145675 | hsa05214 |
| Renal cell carcinoma | PIK3R1, ETS1 | 6.76 | ENSG00000145675 ENSG00000134954 | hsa05211 |
| VEGF signaling pathway | PIK3R1, NFAT5 | 6.66 | ENSG00000145675 ENSG00000102908 | hsa04370 |
| Melanoma | E2F2, PIK3R1 | 6.46 | ENSG00000007968 ENSG00000145675 | hsa05218 |
| Pancreatic cancer | E2F2, PIK3R1 | 6.37 | ENSG00000007968 ENSG00000145675 | hsa05212 |
| Small cell lung cancer | E2F2, PIK3R1 | 5.4 | ENSG00000007968 ENSG00000145675 | hsa05222 |
| Colorectal cancer | FOS, PIK3R1 | 5.4 | ENSG00000170345 ENSG00000145675 | hsa05210 |
| ErbB signaling pathway | CBL, PIK3R1 | 5.27 | ENSG00000110395 ENSG00000145675 | hsa04012 |
| Prostate cancer | E2F2, PIK3R1 | 5.2 | ENSG00000007968 ENSG00000145675 | hsa05215 |
| Toll-like receptor signaling pathway | FOS, PIK3R1 | 4.52 | ENSG00000170345 ENSG00000145675 | hsa04620 |
| Cell cycle | WEE1, E2F2 | 4.01 | ENSG00000166483 ENSG00000007968 | hsa04110 |
| Natural killer cell mediated cytotoxicity | PIK3R1, NFAT5 | 3.7 | ENSG00000145675 ENSG00000102908 | hsa04650 |
| Ubiquitin mediated proteolysis | SOCS1, CBL | 3.43 | ENSG00000185338 ENSG00000110395 | hsa04120 |
