# Supplementary material for: Inflammation triggers specific microRNA profiles in human adipocytes and macrophages and in their supernatants
Source: Clin Epigenetics. 2015 Apr 24;7(1):49. doi: 10.1186/s13148-015-0083-3 (PMC4413548; doi:10.1186/s13148-015-0083-3)
Supplement: Additional file 3: Figure S1. — Workflow diagram. Figure S2. Insulin signaling pathway showing the involvement of CBL, SOCS1, and PIK3R1 (yellow boxes). Their post-transcriptional regulation may be accomplished by both miR-221 and miR-155. Figure S3. SOCS1 and PIK3R1 (yellow boxes) in the context of type II diabetes and impaired glucose intake. [file 13148_2015_83_MOESM3_ESM.ppt]

## Slide 1
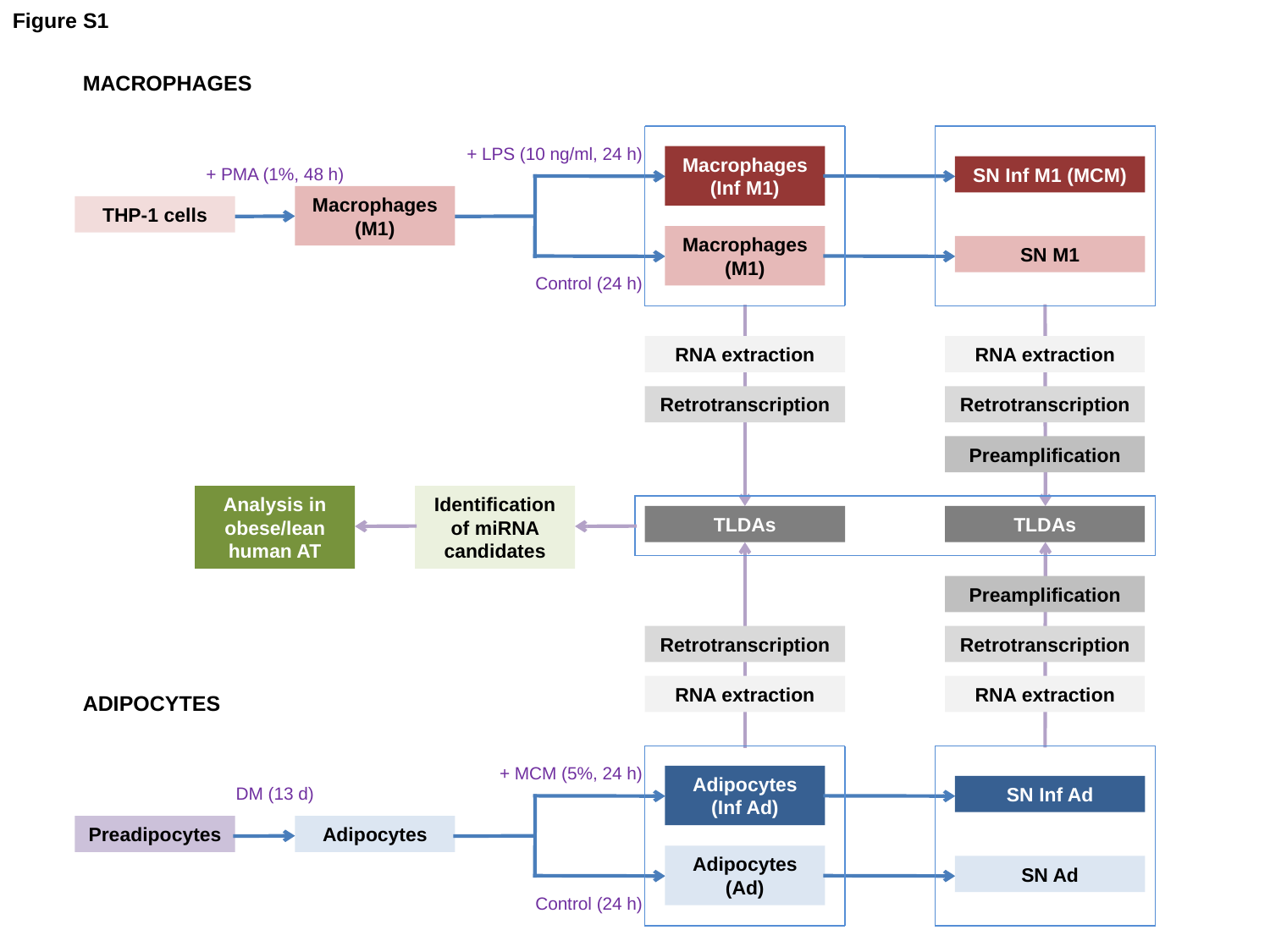

Figure S1
MACROPHAGES
+ LPS (10 ng/ml, 24 h)
Macrophages (Inf M1)
+ PMA (1%, 48 h)
SN Inf M1 (MCM)
Macrophages (M1)
THP-1 cells
Macrophages (M1)
SN M1
Control (24 h)
RNA extraction
RNA extraction
Retrotranscription
Retrotranscription
Preamplification
Analysis in obese/lean human AT
Identification of miRNA candidates
TLDAs
TLDAs
Preamplification
Retrotranscription
Retrotranscription
RNA extraction
RNA extraction
ADIPOCYTES
+ MCM (5%, 24 h)
Adipocytes
(Inf Ad)
DM (13 d)
SN Inf Ad
Preadipocytes
Adipocytes
Adipocytes
(Ad)
SN Ad
Control (24 h)

## Slide 2
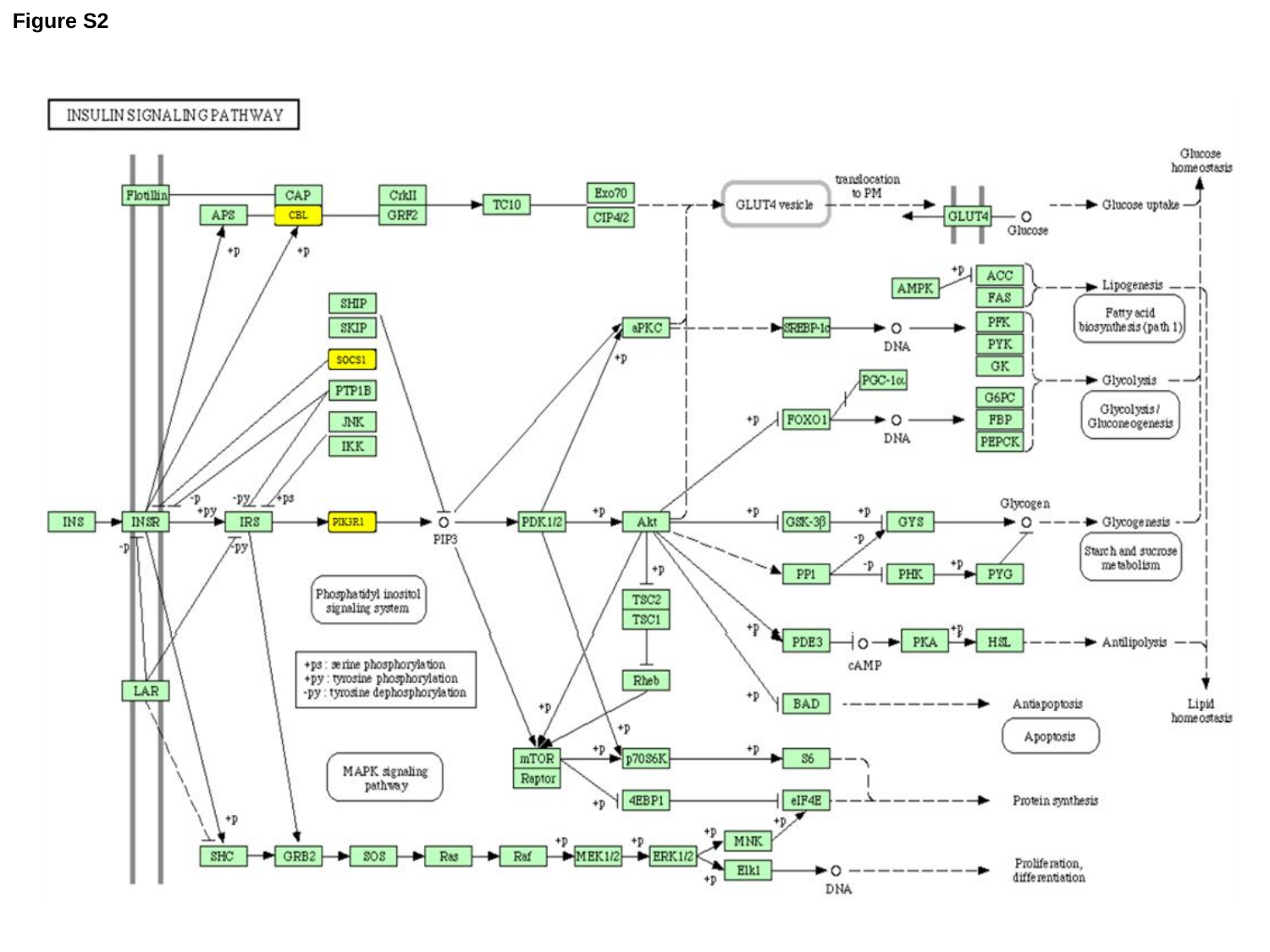

Figure S2

## Slide 3
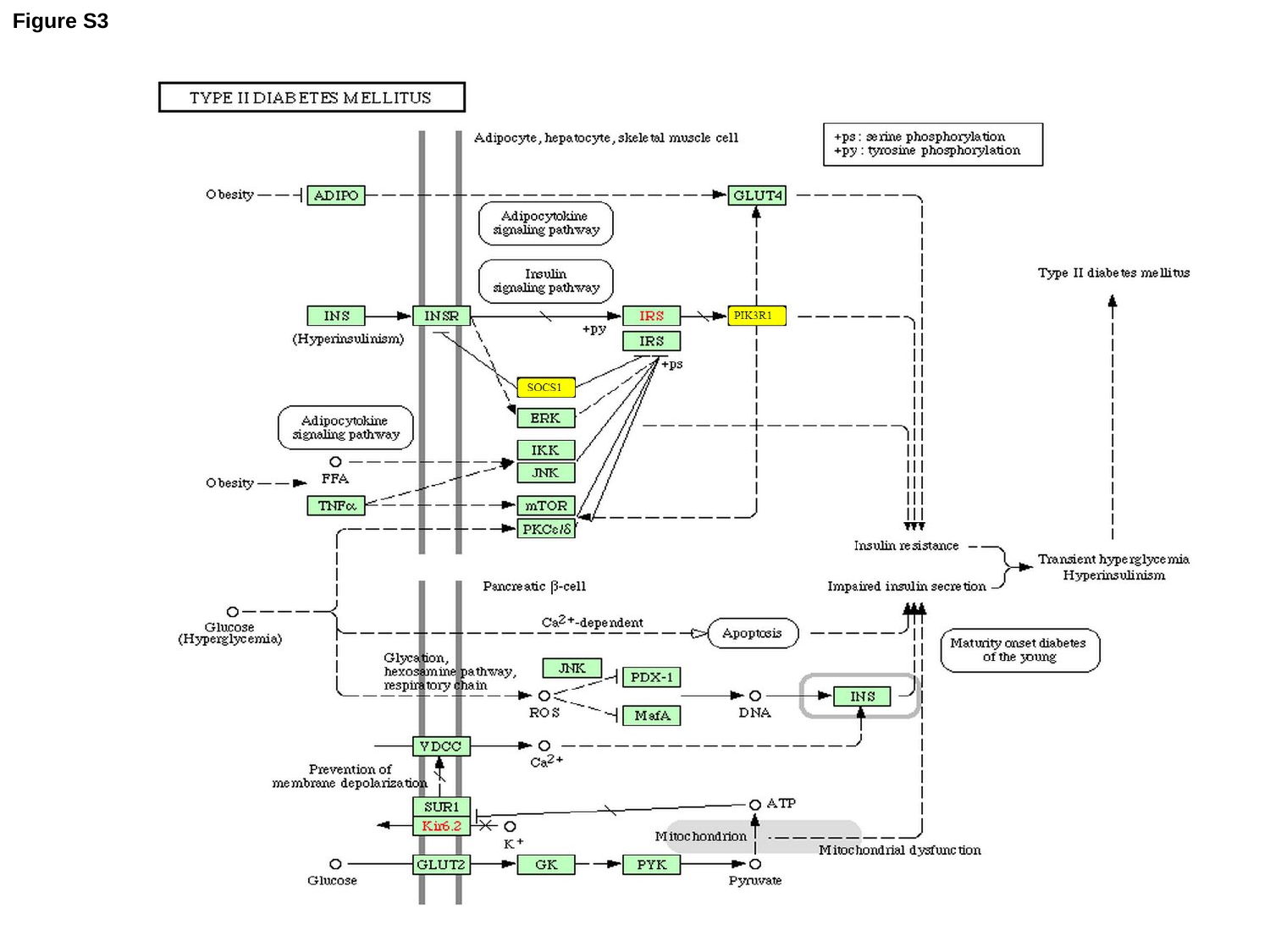

Figure S3
